# Functional Neuroimaging Distinguishes Posttraumatic Stress Disorder from Traumatic Brain Injury in Focused and Large Community Datasets

**DOI:** 10.1371/journal.pone.0129659

**Published:** 2015-07-01

**Authors:** Daniel G. Amen, Cyrus A. Raji, Kristen Willeumier, Derek Taylor, Robert Tarzwell, Andrew Newberg, Theodore A. Henderson

**Affiliations:** 1 Department of Research, Amen Clinics, Inc., Costa Mesa, California, United States of America; 2 Department of Radiology, University of California Los Angeles Medical Center, Los Angeles, California, United States of America; 3 Department of Psychiatry, Faculty of Medicine, University of British Columbia, Lions Gate Hospital, North Vancouver, British Columbia, Canada; 4 Department of Radiology, Thomas Jefferson University, Philadelphia, Pennsylvania, United States of America; 5 The Synaptic Space, Denver, Colorado, United States of America; 6 The International Society of Applied Neuroimaging, Denver, Colorado, United States of America; Univ of Toledo, UNITED STATES

## Abstract

**Background:**

Traumatic brain injury (TBI) and posttraumatic stress disorder (PTSD) are highly heterogeneous and often present with overlapping symptomology, providing challenges in reliable classification and treatment. Single photon emission computed tomography (SPECT) may be advantageous in the diagnostic separation of these disorders when comorbid or clinically indistinct.

**Methods:**

Subjects were selected from a multisite database, where rest and on-task SPECT scans were obtained on a large group of neuropsychiatric patients. Two groups were analyzed: Group 1 with TBI (n=104), PTSD (n=104) or both (n=73) closely matched for demographics and comorbidity, compared to each other and healthy controls (N=116); Group 2 with TBI (n=7,505), PTSD (n=1,077) or both (n=1,017) compared to n=11,147 without either. ROIs and visual readings (VRs) were analyzed using a binary logistic regression model with predicted probabilities inputted into a Receiver Operating Characteristic analysis to identify sensitivity, specificity, and accuracy. One-way ANOVA identified the most diagnostically significant regions of increased perfusion in PTSD compared to TBI. Analysis included a 10-fold cross validation of the protocol in the larger community sample (Group 2).

**Results:**

For Group 1, baseline and on-task ROIs and VRs showed a high level of accuracy in differentiating PTSD, TBI and PTSD+TBI conditions. This carefully matched group separated with 100% sensitivity, specificity and accuracy for the ROI analysis and at 89% or above for VRs. Group 2 had lower sensitivity, specificity and accuracy, but still in a clinically relevant range. Compared to subjects with TBI, PTSD showed increases in the limbic regions, cingulum, basal ganglia, insula, thalamus, prefrontal cortex and temporal lobes.

**Conclusions:**

This study demonstrates the ability to separate PTSD and TBI from healthy controls, from each other, and detect their co-occurrence, even in highly comorbid samples, using SPECT. This modality may offer a clinical option for aiding diagnosis and treatment of these conditions.

## Introduction

Traumatic brain injury (TBI) and posttraumatic stress disorder (PTSD) are complex, commonly comorbid disorders in which clinical symptoms often overlap, creating challenges in diagnosis and treatment [1–3]. Advanced neuroimaging techniques are providing insights into underlying pathological and physiological changes, and biomarker studies offer the potential to differentiate these disorders at acute stages, when interventions have the greatest potential to yield effective outcomes [4, 5]. Structural imaging in acute TBI is indicated for the identification of skull fractures, contusions and bleeds, but computed tomography (CT) or magnetic resonance imaging (MRI) typically do not demonstrate the subtle abnormalities associated with TBI including perfusion deficits, diffuse axonal injury and alterations in functional anatomical connections [6]. As mild TBI often goes undetected using conventional structural imaging [7], the use of functional imaging techniques, including single photon emission computed tomography (SPECT) have demonstrated a greater sensitivity and specificity for identifying mild TBI [8]. The most commonly observed regions affected are the orbitofrontal cortex, temporal poles, and anterior cingulum [8], which correlate with cognitive and psychiatric symptomology. In contrast, imaging of PTSD has revealed volumetric and perfusion changes in the amygdala [9], corpus callosum [10], insula [11], anterior cingulum [12–14] and hippocampus [15, 16]. Given the overlapping symptomology between TBI and PTSD and the high prevalence of these disorders within our active duty and post-deployment U.S. military personnel [17, 18] and civilian population [19, 20], there is a need to identify diagnostic tools that can clearly distinguish these disorders, so patients may be directed toward appropriate treatment. Research into the use of SPECT has demonstrated its clinical utility for both the improved detection of TBI [8, 21–23] and the delineation of the neural circuitry underlying PTSD [24–31], offering the potential of this modality to identify functional biomarkers useful in differential diagnosis. The question remains as to whether SPECT can distinguish PTSD from TBI or the comorbid presence of both. The ability to address this question is complicated by the differences between these conditions in military versus general populations which may be due to mechanisms of injury in addition to other factors.

### TBI and PTSD in Military Personnel

Traumatic brain injury (TBI), especially from blast and blunt force trauma, has been designated as the “signature wound” of the Iraq and Afghanistan wars [32, 33]. The prevalence of these brain disorders in active duty U.S. military personnel is on the rise, with the Department of Defense reporting 307,283 diagnosed cases of TBI from 2000–2014 [17] and the Congressional Research Service reporting 103,792 diagnosed cases of PTSD from 2000–2012 [18]. The economic costs to society for treatment of PTSD and TBI are significant, with the Rand Corporation estimating an annual cost for TBI between $591 and $910 million. Within the first two years after returning from deployment, they estimate that costs associated with PTSD and major depression for 1.6 million service members range between $4.0 to $6.2 billion [34].

The co-occurrence of these two disorders among military personnel also is quite frequent. Service members exposed to TBI have been shown to have a higher incidence of PTSD [35–37]. An observational study by Taylor et al. reported 73% of service members met the criteria for PTSD [38]. The RAND survey of returning combat veterans also reported that 50% had witnessed the death or injury of a friend, 10% had been injured themselves, and over 19% had symptoms consistent with PTSD [34]. Hoge et al. found that 90% of combatants had experienced a traumatizing event [35]. Soldiers who experience blast-related TBI are at greater than double the risk for developing PTSD [36]. Over 400,000 military personnel and veterans have been diagnosed with PTSD or TBI since 2001 [17, 18], and many have been diagnosed with both. Indeed, the overlap of these two populations has been estimated at 33% [34, 35] to 42% [39] among veterans.

### TBI and PTSD in Civilians

Among the U.S. civilian population, approximately 7.7 million suffer from PTSD [19]. TBI is also quite prevalent, with 2.5 million annual visits to emergency rooms for suspected TBI [40]. Research suggests that long-term consequences of seemingly innocuous head injuries may be significant [41, 42], and it is now understood that repetitive TBI, as occurs in sport, can lead to long-term morbidity [43–48].

The co-occurrence of TBI and PTSD in civilian populations is less clearly delineated. Studies among civilians with TBI indicate that 49% develop a new psychiatric illness in the year subsequent to injury [49–51]. Victims of head injury from motor vehicle accidents also have a higher rate of PTSD compared to those with orthopedic injuries. Bombardier and colleagues reported that 44% of victims of TBI related to assault progressed to meeting diagnostic criteria for PTSD, but that among a cohort of civilians with TBI from any cause only 11% developed PTSD [52]. Research on the co-occurrence of these two disorders is scant with a PubMed search revealing only five references on the topic.

### Biomarkers to Differentiate TBI from PTSD

Given 1) the heterogeneous nature of TBI, 2) the fact that mild TBI, which is most common, is less likely to yield obvious, specific chronic symptoms, 3) the reliance of diagnosis on self-report, and 4) the overlap of physical and psychological symptoms between PTSD and TBI, biomarkers to accurately diagnose these disorders would be a welcome adjunct to clinical acumen. Moreover, while TBI symptoms can resolve over time, a significant proportion of cases develop a persistent post-concussive syndrome (PCS) [3]. Some symptoms of PCS [53, 54] overlap with those of PTSD and can include: headache, dizziness, irritability, memory impairment, slowed reaction time, fatigue, sleep disturbances, sensitivity to light and noise, impulsivity, anxiety and depressive symptoms [2, 3, 39, 46–48, 55–60]. Accurately distinguishing TBI from PTSD can regularly be a clinical challenge. Recollection of traumatic events, particularly if assessment occurs after significant time has elapsed, can be inconsistent [61, 62]. The treatments for PTSD and TBI/PCS are different, therefore, reliably separating them, as well as identifying cases in which both are present, emerges as a genuine diagnostic need [56, 59, 63]. To our knowledge, no widely accepted biomarker to distinguish TBI from PTSD has been reported.

### Neuroimaging in TBI

There are a variety of neuroimaging modalities available which provide significant clinical utility in the context of TBI and PTSD. CT and MRI are used to measure changes in anatomical or physiological parameters of TBI (hemorrhage, edema, vascular injury, intracranial pressure), but for most cases of mild TBI, CT and MRI often show no abnormalities [7]. Diffusion tensor imaging (DTI) has been used to detect axonal injury for mild to moderate TBI, but results are inconsistent, highly dependent upon technique, and require further investigation [36]. Functional MRI (fMRI) is often used to differentiate TBI from control groups [64] and has been used to study activation patterns in patients with TBI [65]. Fluorodeoxyglucose positron emission tomography (FDG-PET) measures glucose uptake and metabolism and is used to detect subtle changes in brain function from TBI that are not observed with structural imaging modalities like CT or MRI [66].

Brain perfusion SPECT is used to measure cerebral blood flow and activity patterns and is indicated for the evaluation of TBI in the absence of anatomical findings [67]. A recent review of three decades of research by Raji and colleagues concluded that perfusion SPECT for TBI 1) has improved lesion detection compared to CT/MRI; 2) helps to predict clinical outcomes; and 3) can help direct treatment. Based on their review, the authors suggest that SPECT should be part of a clinical evaluation in the diagnosis and management of TBI [8]. This review cited 19 longitudinal studies that demonstrated Level II A evidence (i.e., evidence from at least one controlled trial without randomization) for perfusion SPECT in identifying lesions in the clinical setting of TBI [8, 68–71]. SPECT has high sensitivity in TBI cases [21–23, 68]. Jacobs et al. found a 91% sensitivity < 3 months after injury and 100% thereafter and strong (96% to 100%) negative predictive value, thus indicating that a negative perfusion SPECT scan is a reliable indicator of a positive outcome for head injury [68].

### Neuroimaging in PTSD

Brain activation studies have been performed to identify the underlying circuits in PTSD using PET, fMRI and SPECT [24–27, 72–74]. A recent meta-analysis of 19 imaging studies using a symptom provocation paradigm showed that PTSD patients have significant activation of the mid-line retrosplenial cortex and precuneus when presented with trauma-related stimuli [75]. Furthermore, the relationship between TBI and PTSD has been studied with functional imaging using FDG-PET in veterans with mild TBI and/or PTSD compared to community volunteers [76].

Network-based studies of PTSD using fMRI reviewed by Peterson report a positive association between default mode network (DMN) activity and PTSD severity [77]. This correlation was also reported by Lanius [78]. The DMN is postulated as a circuit active during the resting state, involving the inferior orbital frontal cortex, anterior and posterior cingulum, hippocampus, precuneus, superior parietal lobe, and the angular gyrus [79]. If a hyperactive DMN is causal in severe PTSD, this may reflect a failure of DMN regulation which manifests clinically as diminished affect regulation.

Perfusion SPECT also has been investigated in the evaluation of PTSD, and preliminary data suggest it has a potential role in distinguishing PTSD from TBI [25–28]. For example, increased perfusion of the caudate has been associated with PTSD [28]. A small study using both perfusion SPECT and FDG PET showed that women with PTSD had significant decreases in perfusion in the left hippocampus and in the basal ganglia, and lower cerebral glucose metabolism in the left hippocampus and the superior temporal and precentral gyri than in the control group [80]. Another SPECT study showed that compared to controls, PTSD patients had increased cerebral blood flow in the limbic regions along with decreased perfusion in the superior frontal, parietal, and temporal regions [81].

### Neuroimaging to Differentiate TBI, PTSD and the Comorbid Condition

While previous studies have explored the relationship between PTSD and TBI using neuroimaging [5, 76, 82, 83], to the best of our knowledge, no study has identified imaging biomarkers differentiating PTSD from TBI using brain SPECT imaging In addition, one barrier to identifying neuroimaging biomarkers for psychiatric disorders has been the lack of sufficiently large-scale studies [84]. In this retrospective study, two groups were analyzed; i) a small, well-defined group with the diagnosis of TBI and/or PTSD at one clinical site closely matched for demographics and comorbidity compared to a healthy dataset, and ii) a large, generalized group of all TBI and/or PTSD patients regardless of comorbidity across multiple-sites which were also compared to healthy controls. Both region of interest (ROI) and visual readings (VRs) were analyzed to assess the diagnostic accuracy in using SPECT to better assess and diagnose these conditions.

## Methods

### Study Subjects

This study was conducted in accordance with the STARD guidelines (http://www.stard-statement.org/). All subjects were obtained for retrospective analysis from a large multisite psychiatric database, involving 20,746 patients who came for evaluation of psychiatric and/or neurological conditions to one of nine outpatient clinics (Newport Beach, Costa Mesa, Fairfield, and Brisbane, CA, Tacoma and Bellevue, WA, Reston, VA, Atlanta, GA and New York, NY) from 1995–2014. Diagnoses were made by board certified or eligible psychiatrists, using all of the data available to them, including detailed clinical history, mental status examination and DSM-IV or V criteria, consistent with the current standard of care.

The retrospective data analysis for this study was approved by the IRB IntegReview (http://www.integreview.com/) (IRB #004) and the healthy subjects were obtained in a separate study as approved by the Western Institutional Review Board (WIRB # 20021714). Written informed consent was obtained from all healthy subjects and data mining of anonymous clinical data was sanctioned in accordance with 45 CFR 46.101(b)(4).

Included in the database are n = 116 healthy adult volunteers who had resting state and on-task SPECT studies. The exclusion criteria for the healthy subjects were: 1) current or past evidence of psychiatric illnesses as determined by clinical history, mental status examinations, and the Structured Clinical Interview for Diagnosis for DSM-IV; 2) current reported medical illnesses or medication; 3) history of brain trauma; 4) current or past drug or alcohol abuse; 5) first degree relative with a psychiatric illness.

**Fig 1:**
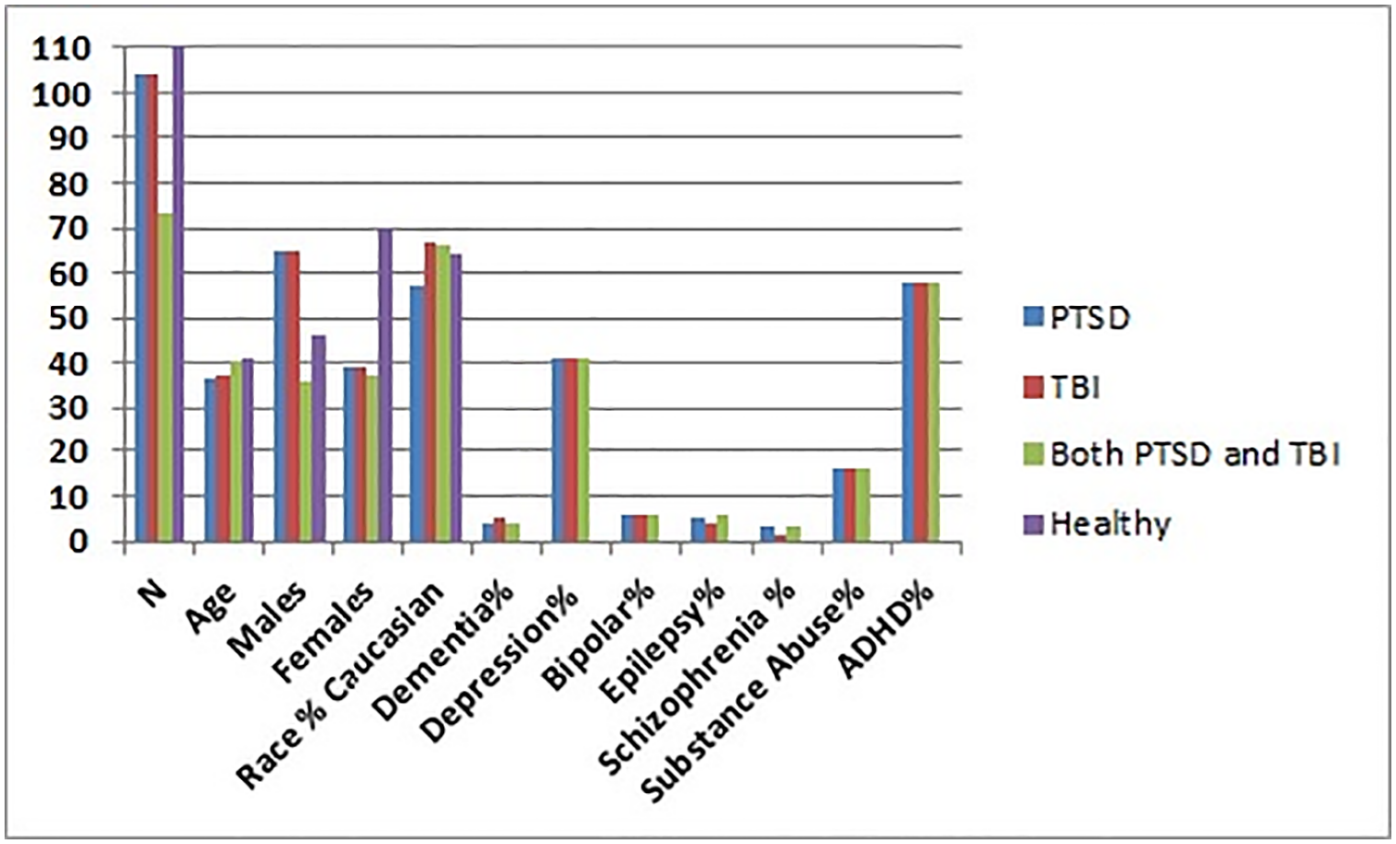
Proportional Demographics for Group 1. Demographics of Group 1 with number of patients, number of males, number of females and age expressed in absolute numbers, and all other values expressed as percentages.

Two groups were extracted from the larger database for analysis. Group 1 (n = 397) is described in Table 1 and Fig 1.

**Table 1:** Subject Demographics for Group 1.

Variable	PTSD (n = 104)	TBI (n = 104)	Both PTSD and TBI (n = 73)	Healthy (n = 116)
Age	36.7 ± 12.9	37.2 ±12.9	40.7 ± 13.8	41.4 ± 17.9
Gender(M/F)	65/39	65/39	36/37	46/70
Race% Caucasian	57	67	66	64
Dementia%	4	5	4	0
Depression%	41	41	41	0
Bipolar%	6	6	6	0
Epilepsy%	5	4	6	0
Schizophrenia%	3	1	3	0
Substance Abuse%	16	16	16	0
ADHD%	58	58	58	0

Group 1 represents a select cohort from the Newport Beach site matched closely by demographics and co-morbidities, including the healthy cohort. In this group, variance due to comorbid diagnoses was minimized and Ns were matched closely (n = 104 for TBI or PTSD, n = 73 for TBI + PTSD, n = 116 for controls) as the main inclusion criteria. The primary selection criterion for TBI in clinical trials is the GCS, which is used to assess the level of consciousness following a TBI [85]. It rates a patient’s level of consciousness based on the ability to open his or her eyes, talk and move. As this was a retrospective chart review and subjects were not assessed for TBI at the time of injury, we were unable to use the GCS as an assessment of injury severity. Therefore, subjects were classified according to injury severity categories of mild, moderate or severe based on the Department of Defense Clinical Practice Guidelines [86]. Further classification included type of injury (blunt, penetrating, unknown) and mode of injury (accident, assault, fall, sport, accident, unknown) as shown in Tables 2 and 3.

**Table 2:** TBI Classification for Group 1.

Severity	Type of Injury	Mode of Injury	Number of Patients
Mild	blunt	accident	18
	blunt	assault	5
	blunt	fall	14
	blunt	sports	17
	penetrating	accident	2
	unknown	unknown	9
Moderate	blunt	accident	4
	blunt	assault	1
	blunt	fall	5
	blunt	sports	1
Severe	blunt	accident	13
	blunt	assault	1
	blunt	fall	2
	blunt	sports	2
Unknown Severity	blunt	accident	1
	blunt	assault	1
	blunt	fall	2
	blunt	sports	1
	unknown	unknown	5

**Table 3:** TBI Classification with Comorbid PTSD for Group 1.

Severity	Type of Injury	Mode of Injury	Number of Patients
Mild	blunt	accident	20
	blunt	assault	5
	blunt	fall	8
	blunt	sports	8
	blunt	unknown	1
	unknown	accident	1
	unknown	unknown	4
Moderate	blunt	accident	3
	blunt	assault	1
	blunt	fall	1
	blunt	sports	2
	penetrating	assault	1
Severe	blunt	accident	5
	blunt	assault	1
	blunt	fall	1
	blunt	sports	1
Unknown Severity	blunt	accident	3
	blunt	assault	2
	blunt	fall	1
	blunt	sports	2
	unknown	accident	1
	unknown	unknown	1

Of the 104 patients with TBI only, 65 were classified as mild, 11 moderate and 18 severe based on neuroimaging data and clinical interview. Ten patients could not be categorized based on information provided in the chart. Of these 10 patients, 8 showed abnormal findings on SPECT scans indicating trauma: four subjects were diagnosed with temporal lobe dysfunction; one subject with prefrontal lobe dysfunction and temporal lobe dysfunction; one subject with cerebellar dysfunction, parietal lobe dysfunction and temporal dysfunction; one subject with frontal lobe syndrome and prefrontal lobe dysfunction; and one subject with post-concussion syndrome. Of the 73 patients with TBI+PTSD, 47 were classified as mild, 8 moderate and 8 severe. Ten patients could not be categorized based on information provided in the chart. Of these 10 subjects, 6 had abnormal findings on SPECT scans indicating trauma: one patient showed frontal lobe syndrome, limbic system dysfunction, parietal lobe dysfunction and temporal dysfunction; one subject was diagnosed with post-concussion syndrome; one subject showed occipital lobe hyperperfusion, parietal lobe dysfunction and temporal dysfunction; one subject showed prefrontal lobe and temporal dysfunction; and two subjects showed frontal lobe syndrome and temporal dysfunction.

The patients in the TBI group had a chart diagnosis of intracranial injury with a brief or extended loss of consciousness (n = 62) or concussion (n = 42). The patients with PTSD met the DSM-IV criteria. Patients in the subgroup with TBI were compared to those with PTSD, those with TBI+PTSD, and to healthy controls. Similarly, each of the other subgroups was compared to the remaining subgroups in the method described below.

**Fig 2:**
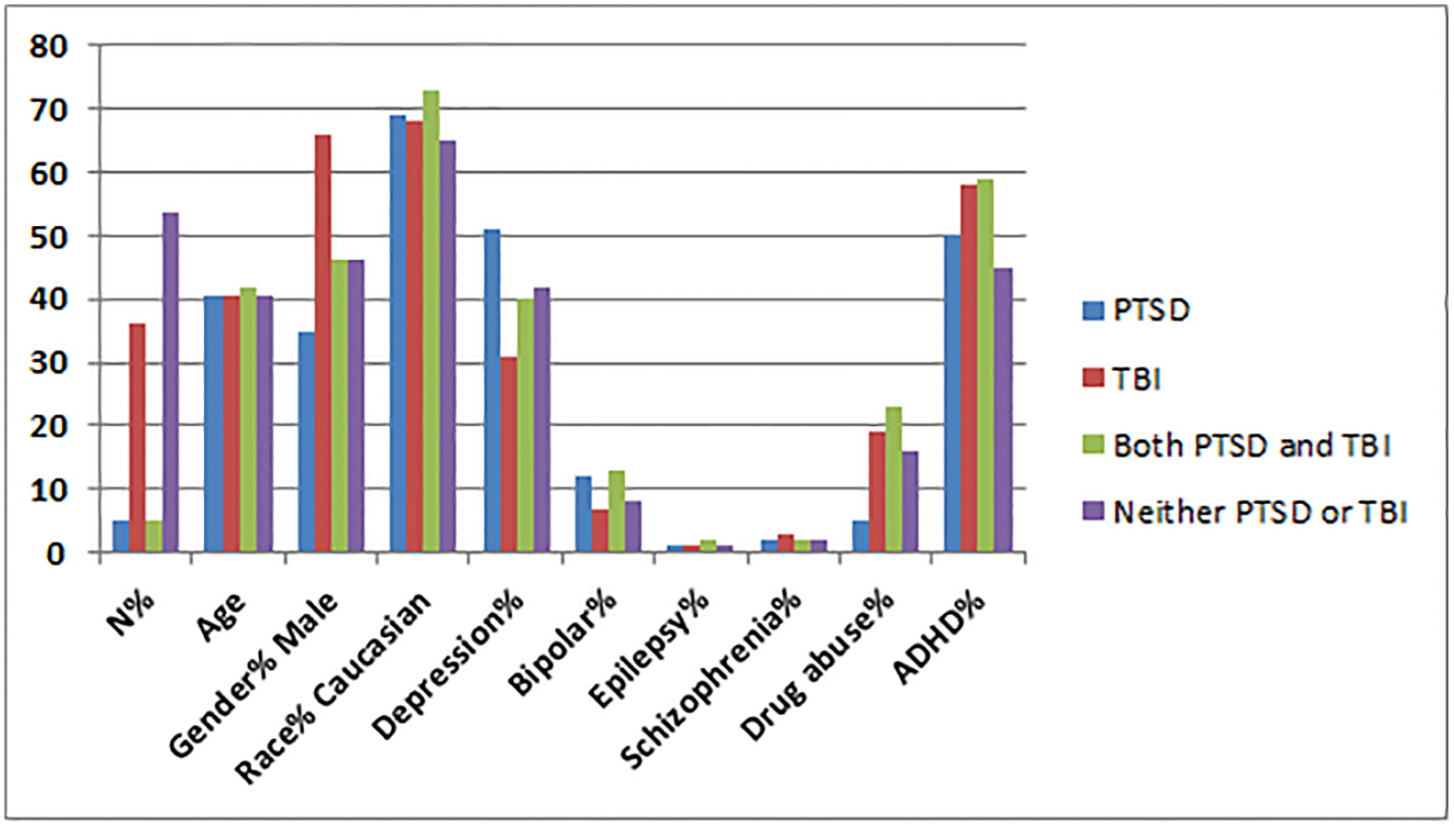
Proportional Demographics for Group 2. Demographics of Group 2 with number of patients with age expressed in years and all other numbers expressed as percentages.

Group 2 consists of a generalized group with much larger cohorts of patients in each diagnostic area, but unmatched for demographics or comorbidity across all sites. The patients in the TBI and PTSD groups both had a chart diagnosis for their specific disorders. Group 2 reflects the full range of psychiatric co-morbidities across the larger cohorts (n = 7,505 for TBI, n = 1,077 for PTSD, n = 1,017 for TBI+PTSD, n = 11,147 which do not include TBI or PTSD). Group 2 is described in Table 4 and Fig 2. Each subgroup was compared to the other subgroups as described below.

**Table 4:** Subject Demographics for Group 2.

Variable	PTSD (n = 1077)	TBI (n = 7505)	Both PTSD and TBI (n = 1017)	Neither PTSD or TBI (n = 11147)
Age	40.7 ± 13.9	40.5 ± 15.6	41.9 ± 13.7	40.6 ± 16.5
Gender% Male	35	66	46	46
Race% Caucasian	69	68	73	65
Depression%	51	31	40	42
Bipolar%	12	7	13	8
Epilepsy%	1	1	2	1
Schizophrenia%	2	3	2	2
Drug abuse%	5	19	23	16
ADHD%	50	58	59	45

### SPECT Imaging Acquisition

All SPECT scans were performed using a high resolution Picker (Philips) Prism XP 3000 triple-headed gamma camera (Picker Int. Inc., Ohio Nuclear Medicine Division, Bedford Hills, OH, USA) with low energy high resolution fan beam collimators. SPECT was performed as previously described [87, 88]. For each procedure, an age- and weight-appropriate dose of 99mTc–hexamethylpropyleneamine oxime (HMPAO) SPECT was administered intravenously at rest and while performing a cognitive task. For the rest scans, patients were injected while they sat in a dimly lit room with eyes open. Patients were scanned approximately 30 minutes after injection. For the on-task scans, patients were injected three minutes after starting the Conners Continuous Performance Test (Conners Continuous Performance Test, CCPT-II, Multi-Health Systems, Toronto, Ontario). Approximately 30 minutes after the injection, subjects were scanned. Data was acquired in 128x128 matrices, yielding 120 images per scan with each image separated by three degrees spanning 360 degrees. The original image matrix obtained at 128x128x29 with voxel sizes of 2.16mm x 2.16mm x 6.48mm were transformed and resliced to a 79x95x68 matrix with voxel sizes of 2mm x 2mm x 2mm consistent with the MNI template. Images were smoothed using an 8mm FWHM isotropic Gaussian kernel. The slice thickness was 6mm. A low pass filter was applied with a high cutoff. Chang attenuation correction was performed [89]. Transaxial slices oriented horizontal to the AC-PC line were created along with coronal and sagittal images (6.6mm apart, unsmoothed).

### SPECT Region of Interest Analysis

ROI counts were derived from the anatomical regions in the AAL atlas [90]. These quantitative ROI metrics were in no way used to aid in the clinical diagnosis of PTSD or TBI. To account for outliers, T-score derived ROI count measurements were derived using trimmed means [91] that are calculated using all scores within the 98% confidence interval (-2.58 < Z < -2.58). The ROI mean for each subject and the trimmed mean for the sample are used to calculate T with the following formula: T = 10*((subject ROI_mean - trimmed regional_avg)/trimmed regional_stdev)+50.

### SPECT Visual Reading Analysis

All scans were read visually by experienced SPECT readers (6–23 years of experience). Methods for visual readings have been fully described in previously published work [87, 88]. Briefly, 14 cortical regions of interest (ROIs) in orthogonal planes were visually inspected and rated using the Mai Atlas of the Human Brain [92]: left and right prefrontal poles; left and right inferior orbits; left and right anterior/lateral PFC; left and right midlateral PFC; left and right posterior frontal region; left and right parietal lobes; and left and right occipital lobes. In like manner, the left and right cerebellar hemispheres and vermis were rated. In addition, subcortical regions were rated, including the dorsal, genu and ventral aspect of the anterior cingulate gyrus; middle and posterior cingulate; left and right insula; left and right caudate nuclei; and left and right putamen. Raters did not have access to detailed clinical information, but did know age, gender, medications, and primary presenting symptoms (ex. depressive symptoms, apathy, etc.). The following nonlinear scheme was used to visually rate rCBF: activity rated above the top 95% was assigned a score of 4+; 91%-95% was scored 3+; 86%-90% was scored 2+; 81%-85% was scored 1+; 61%-80% was scored 0; 56%-60% was scored–1; 51%-55% was scored–2; 46%-50% was scored–3; and 41%-45% was scored–4, resulting in a rating scale ranging from +4 to -4 in a-point intervals.

### Statistical Analyses

The majority of statistical analyses were performed using Statistical Package for Social Science (SPSS, version 22, IBM, Armonk, NY) with additional 10-fold cross validation analysis performed in R (http://www.r-project.org/). Data were analyzed first at UCLA (CAR) with analyses repeated and results verified independently at the Amen Clinics (DGA) and Thomas Jefferson University (AN). Multiple imputation analysis did not identify any significant missing data (<10%). In doing the analyses, the following steps were invoked: First, binary logistic regression models were built using either rest ROIs, rest VRs, on-task ROIs, or on-task VRs as predictor variables. Cerebellar and vermis regions were averaged to carefully optimize subject to variable ratios. Paired comparisons between the groups described in Tables 1 and 4 were performed. Covariates in the analysis were age, gender, race and psychiatric co-morbidities listed in Tables 1 and 4. For Group 2, an additional covariate of study site ID was included in the analysis. Predicted probabilities from binary logistic regression models were then inputted into a Receiver Operating Characteristic (ROC) analysis to identify sensitivity, specificity, and accuracy in delineating between the various clinical groups with 95% confidence intervals Finally, a One Way ANOVA with Least Square Differences (LSD) for correcting for multiple comparisons was done to identify the most diagnostically important regions in separating PTSD from TBI. This analysis was also done to determine if increases or decreases in these diagnostically important regions were the main predictors of diagnostic utility of the SPECT regions tested. 10-fold cross validated models [93] were compared against the main models to assess for performance measure stability.

## Results

For Group 1, rest and on-task ROIs and VRs show significant separations from PTSD, TBI and combined conditions. The non-comorbid group separates 100% with the method described for the ROI analysis and above 89% for accuracy for the VRs. The larger comorbid group has lower sensitivity, specificity and accuracy, but these remain in a clinically relevant range (Tables 5 and 6).

**Table 5:** Group 1 ROC Analysis of Task vs. Rest Scans.

Group 1 ROC Analysis (ROI/VR) (%)		TBI from PTSD	PTSD From Co-Occurrence	TBI from Co-Occurrence	TBI from Control	PTSD from Control	Co-Occurrence from Control
Sensitivity on-Task		100/100	100/84	100/100	100/100	100/100	100/100
Sensitivity at Rest		100/86	100/82	100/84	100/100	100/100	100/100
Specificity on-Task		100/100	100/80	100/100	100/100	100/100	100/100
Specificity at Rest		100/81	100/80	100/76	100/100	100/100	100/100
Accuracy on-Task		100/100*	100/90	100/100*	100/100*	100/100*	100/100*
	(p-value, 95% C.I.)		.00, .85-.95				
Accuracy at Rest		100/94	100/92	100/89	100/100*	100/100*	100/100*
	(p-value, 95% C.I.)	.00, .89-.97	.00, .88-.96	.00, .83-.93			

Comparison of Quantitative ROIs with Visual Readings (VR) in Distinguishing TBI from PTSD

* p = .000, 95% C.I. = [1-1]

**Table 6:** Group 2 ROC Analysis of Task vs. Rest Scans.

Group 2 ROC Analysis (ROI/VR) (%)		TBI from PTSD	PTSD from Co-Occurrence	TBI from Co-Occurrence	PTSD from Control	TBI from Control	Co-Occurrence from Control
Sensitivity on-Task		82/80	70/70	70/70	70/70	70/67	70/70
Sensitivity at Rest		80/80	70/70	70/70	70/70	70/70	70/70
Specificity on-Task		60/61	61/61	55/56	58/55	54/58	58/57
Specificity at Rest		62/60	60/62	55/56	54/54	54/54	60/59
Accuracy on-Task		78/78	73/71	68/69	68/67	66/66	70/69
	(p-value, 95% C.I.)	.00, .76-.80/.00, .77-.80	.00, .71-.75/.00, .69-.74	.00, .66-.70/.00, .67-.71	.00, .65-.69/.00, .66-.70	.00, .65-.67/.00, .65-.67	.00, .68-.72/.00, .67-.71
Accuracy at Rest		78/77	72/71	68/68	67/66	67/66	70/69
	(p-value, 95% C.I.)	.00, .77-.80/.00, .75-.79	.00, .69-.74/.00, .69-.74	.00, .66-.70/.00, .66-.70	.00, .65-.69/.00, .65-.68	.00, .66-.68/.00, .65-.67	.00, .69-.72/.00, .67-.71

Comparison Quantitative ROIs with Visual Readings (VR) in Distinguishing TBI from PTSD

The most significant regions separating PTSD from TBI for the Group 1 ROI analysis of rest and on-task scans are: limbic regions (amygdala, hippocampus, anterior, middle and posterior cingulum, and thalamus), anterior cerebellum, basal ganglia (caudate and putamen), insula, areas of the prefrontal cortex (inferior orbits, operculum), and temporal lobes (middle and superior temporal lobes and temporal poles). All PTSD-identifying regions were more active on SPECT when compared across all groups, and the TBI-identifying regions were correspondingly hypoactive (Table 7).

**Table 7:** Regional Increases in rCBF that Differentiate PTSD from TBI using ROI Analysis in Distinguishing TBI from PTSD.

Brain Region	Group 1: TBI vs PTSD At Rest	Group 1: TBI vs PTSD On-Task	Group 2: TBI vs PTSD At Rest	Group 2: TBI vs PTSD On-Task
**Limbic**	Amygdala	Amygdala	Amygdala	Amygdala
	Hippocampus	Hippocampus	Hippocampus	Hippocampus
	Ant Cingulum	Ant Cingulum	Ant Cingulum	Ant Cingulum
	Mid Cingulum	Mid Cingulum	Mid Cingulum	Mid Cingulum
	Post Cingulum	Post Cingulum	Post Cingulum	Post Cingulum
	Thalamus	Thalamus	Thalamus	Thalamus
**Basal Ganglia**	Caudate	Caudate	Caudate	Caudate
	Putamen	Putamen	Putamen	Putamen
**Insula**	Insula	Insula	Insula	Insula
**Prefrontal Cortex**	Inferior Orbits	Inferior Orbits	Inferior Orbits	Inferior Orbits
	Operculum	Operculum	Operculum	Operculum
**Temporal Lobes**	Middle Temporal Lobe	Middle Temporal Lobe	Middle Temporal Lobe	Middle Temporal Lobe
	Superior Temporal Lobe	Superior Temporal Lobe	Superior Temporal Lobe	Superior Temporal Lobe
	Temporal Poles	Temporal Poles	Temporal Poles	Temporal Poles
**Cerebellum**	Anterior Cerebellum	Anterior Cerebellum	Anterior Cerebellum	Anterior Cerebellum

PTSD shows increased rCBF in the limbic centers, basal ganglia, insula, prefrontal cortex, temporal lobes, cerebellum, occipital lobe and parietal lobe as compared to TBI both at rest and during a concentration task in Groups 1 and 2 using ROI analysis. Legend for Abbreviations: Ant = Anterior; Mid = Middle; Post = Posterior; Sup = Superior.

The most significant regions from Group 1 VRs of resting state and on-task scans are: limbic regions (right amygdala, left hippocampus, anterior and middle cingulum, thalamus), cerebellum, basal ganglia (caudate during on-task), right insula at rest, multiple areas of the prefrontal cortex (inferior orbits and anterior lateral prefrontal cortex), and temporal lobes (temporal poles and anterior lateral temporal lobes). All PTSD regions were more active than the TBI regions (Table 8).

**Table 8:** Regional Increases in rCBF that Differentiate PTSD from TBI using Visual Readings (VR) in Distinguishing TBI from PTSD.

Brain Region	Group 1: TBI vs PTSD At Rest	Group 1: TBI vs PTSD On-Task	Group 2: TBI vs PTSD At Rest	Group 2: TBI vs PTSD On-Task
**Limbic**	Amygdala	Amygdala	Amygdala	Amygdala
	Hippocampus	Hippocampus	Hippocampus	Hippocampus
	Ant Cingulum	Ant Cingulum	Ant Cingulum	Ant Cingulum
	Mid Cingulum	Mid Cingulum	Mid Cingulum	Mid Cingulum
	Thalamus	Thalamus	Thalamus	Thalamus
**Basal Ganglia**		Caudate	Caudate	Caudate
**Insula**	Insula			
**Prefrontal Cortex**	Inferior Orbits	Inferior Orbits	Inferior Orbits	Inferior Orbits
	Ant Lateral Prefrontal Cortex	Ant Lateral Prefrontal Cortex	Ant Lateral Prefrontal Cortex	Ant Lateral Prefrontal Cortex
			Pole prefrontal cortex	Pole prefrontal cortex
**Temporal Lobes**	Temporal Poles	Temporal Poles	Temporal Poles	Temporal Poles
	Ant Lateral Temporal Lobe	Ant Lateral Temporal Lobe	Ant Lateral Temporal Lobe	Ant Lateral Temporal Lobe
			Mid Lateral Temporal Lobe	Mid Lateral Temporal Lobe
			Post Lateral Temporal Lobe	Post Lateral Temporal Lobe
**Cerebellum**	Cerebellum	Cerebellum	Cerebellum	Cerebellum
**Occipital Lobe**			Occipital Lobe	Occipital Lobe
**Parietal Lobe**			Parietal Lobe	Parietal Lobe

PTSD shows increased rCBF in the limbic centers, basal ganglia, insula (Group 1 at rest only), prefrontal cortex, temporal lobes, cerebellum, occipital lobe and parietal lobe as compared to TBI both at rest and during a concentration task in Groups 1 and 2 using visual readings. Legend for Abbreviations: Ant = Anterior; Lat = Lateral; Mid = Middle; Post = Posterior

The most significant regions from Group 2 ROI analysis of rest and on-task scans are: limbic regions (amygdala, hippocampus, anterior, middle and posterior cingulum, thalamus), anterior cerebellum, basal ganglia (caudate and putamen), insula, areas of the prefrontal cortex (inferior orbits, operculum), and temporal lobes (middle and superior temporal lobes and temporal poles). All PTSD regions were more active than the TBI regions (Table 7). Group 2 baseline ROI differences are quantified in Table 9.

**Table 9:** Baseline ROI Differences between TBI and PTSD Group 2.

Brain Level	Brain Area	TBI		PTSD		Statistic		
		Mean	STDEV	Mean	STDEV	PTSD-TBI	F	Sig
**Cerebellum**	Cerebellum 3 Left	53.11	7.23	55.64	7.5	2.53	5.98	0.014461
	Vermis 1 2	52.74	7.66	55.38	8.01	2.64	6.55	0.010529
**Frontal Lobe**	Rectus Left	52.25	6.93	54.1	7.53	1.85	4.67	0.030794
	Rectus Right	51.74	6.75	53.64	7.36	1.9	4.03	0.044795
**Insula**	Insula Left	56.67	7.45	59.56	8.01	2.89	6.84	0.008915
	Insula Right	57.18	7.61	60.01	8.24	2.83	4.6	0.032072
**Limbic**	Amygdala Left	51.83	6.93	54.63	7.3	2.8	8.53	0.003509
	Amygdala Right	51.76	7.05	54.43	7.36	2.66	4.38	0.036363
	Cingulum Ant Left	55.26	8.09	58.59	8.48	3.33	9.04	0.002648
	Cingulum Ant Right	51.82	7.54	55.05	7.92	3.23	10.93	0.000952
	Cingulum Mid Left	57.19	7.8	60.17	8.32	2.98	5.48	0.019304
	Cingulum Mid Right	55.1	7.5	58.01	7.9	2.9	5.9	0.015155
	Cingulum Post Left	56.29	8.38	59.32	8.89	3.03	5.41	0.020045
	Hippocampus Left	51.31	6.94	53.98	7.25	2.67	4.04	0.044481
	Hippocampus Right	51.42	7.25	54.3	7.47	2.88	5.43	0.019775
	ParaHippocampal Left	46.88	5.75	49.19	6.23	2.31	15.21	0.000097
	ParaHippocampal Right	50.57	6.42	52.97	6.75	2.39	8.3	0.00398
**Olfactory**	Olfactory Left	52.43	7.1	55.26	7.54	2.83	9.8	0.001748
	Olfactory Right	53.45	7.04	56.21	7.57	2.76	7.84	0.005115
**Parietal**	Precuneus Left	48.71	6.06	50.78	6.37	2.07	4.38	0.036363
**Prefrontal Cortex**	Frontal Inf Orb Left	47.88	5.95	49.45	6.27	1.57	8.31	0.003963
	Frontal Inf Orb Right	44.45	5.63	45.64	5.97	1.19	5.73	0.016679
**Temporal Lobes**	Fusiform Left	52.62	6.31	54.66	6.72	2.05	4.65	0.031073
	Fusiform Right	51.67	6.27	53.66	6.57	1.99	4.13	0.042155
	Heschl Left	58.21	8.03	61.15	8.5	2.95	4.62	0.031683
	Temporal Inf Mid Left	44.24	5.69	45.21	5.96	0.97	7.34	0.006746
	Temporal Inf Mid Right	40.03	5.47	40.51	5.73	0.49	3.91	0.047952
	Temporal Mid Ant Left	49.55	6.22	51.28	6.69	1.74	6.3	0.012082
	Temporal Mid Mid Left	51.54	6.5	53.55	6.79	2.01	4.67	0.030794
	Temporal Pole Sup Left	45.13	5.3	46.82	5.76	1.69	10.6	0.001137
	Temporal Pole Sup Right	42.19	5.08	43.54	5.61	1.35	8.91	0.002853
	Temporal Sup Ant Left	53.35	6.99	56.01	7.43	2.66	7.47	0.006274
	Temporal Sup Mid Left	56.02	7.49	58.81	7.8	2.79	6.15	0.013179

Analysis of variance between shows higher cerebral blood flow in subjects with PTSD compared to TBI patients in the cerebellum, frontal lobes, insular cortex, limbic system, olfactory and parietal lobes, prefrontal cortex, and temporal lobes. Significance is shown by F statistic, difference of the means and p value (all areas with p <0.05). Legend for Abbreviations: Ant = Anterior; Inf = Inferior; Lat = Lateral; Mid = Middle; Orb = Orbital; Post = Posterior; Sup = Superior

The most significant regions from Group 2 VRs of rest and on-task scans are: limbic regions (amygdala, hippocampus, and anterior and middle cingulum), cerebellum, basal ganglia (caudate), occipital and parietal lobes, multiple areas of the prefrontal cortex (inferior orbits, anterior lateral prefrontal cortex and prefrontal pole–only left side at baseline), and temporal lobes (temporal poles and anterior, mid and posterior lateral temporal lobes). All PTSD regions were more active than the TBI regions (Table 8). Analysis included a 10-fold cross validation of the protocol in the larger community sample (Group 2), which confirmed the findings. Table 10 and Table 11 show that the difference in performance between the two models produce the same sensitivity, specificity, and accuracy results in ranges from between less than. 01% and. 11%, which is within the acceptable range as defined by the 95% CI.

**Table 10:** Comparative Performance Measures in a 10-Step Cross Validation in the Group 2 Diagnostic Separation of TBI from PTSD.

Group 2 ROC Analysis (%)	TBI from PTSD	
	ROI	VR
Δ Sensitivity on-Task	-0.29	<0.01
Δ Sensitivity at Rest	-0.29	<0.01
Δ Specificity on-Task	-0.78	<0.01
Δ Specificity at Rest	-1.37	0.11

**Table 11:** Confidence Intervals.

Group 2 ROC Analysis (%)	TBI from PTSD		Confidence Intervals		Within CI	
	ROI	VR	ROI	VR	ROI	VR
Δ Accuracy on-Task	-0.34	0.02	(-0.79,0.77)	(-0.85,0.82)	Y	Y
Δ Accuracy at Rest	-0.34	<0.01	(-0.80,0.76)	(-0.87,0.84)	Y	Y

**Fig 3:**
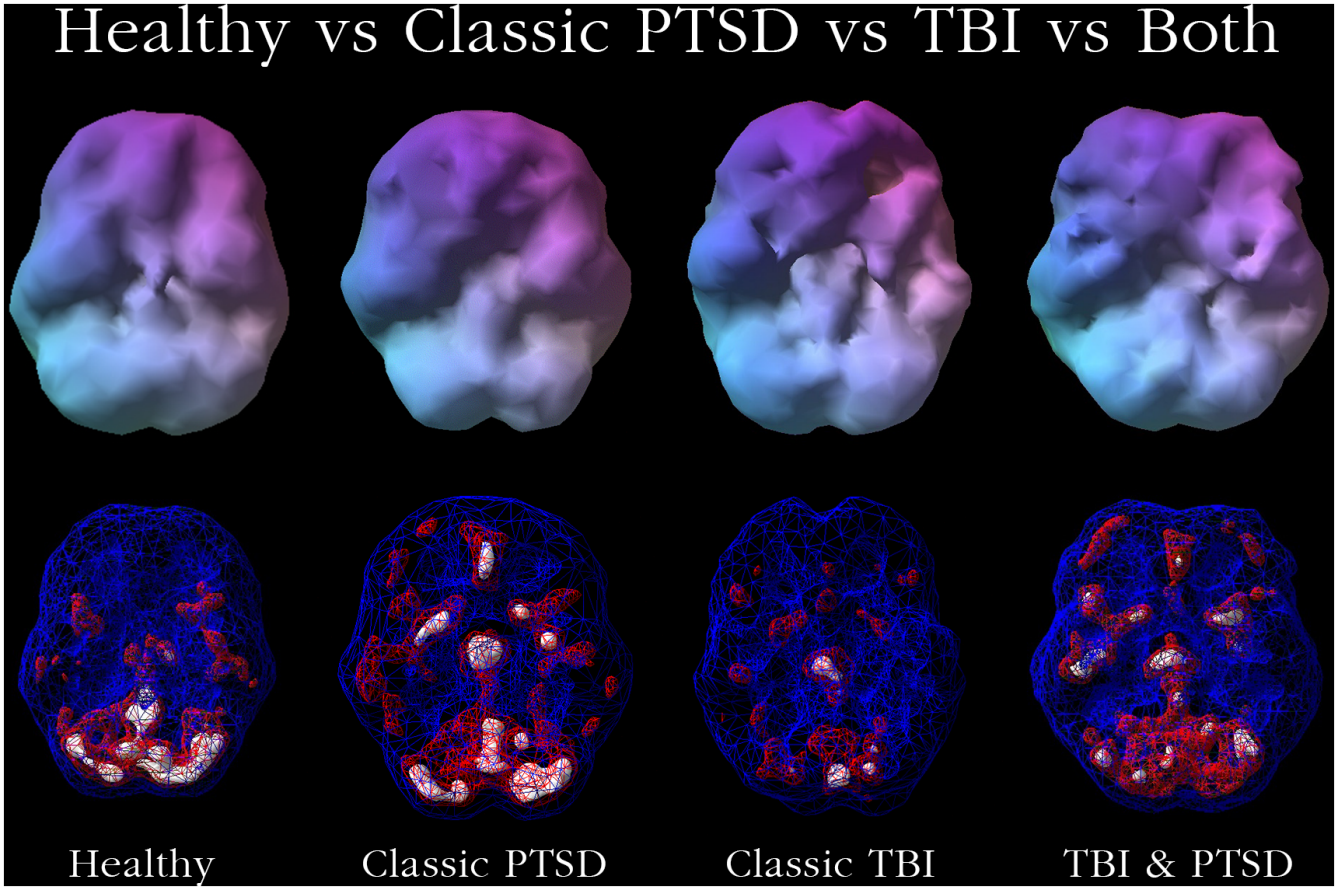
Brain SPECT Images of Healthy, PTSD, TBI and PTSD Co-morbid with TBI Perfusion Patterns. Top row, underneath surface scans, threshold set at 55%, looking at top 45% of brain perfusion. Bottom row, underneath active scans where blue = 55%, looking at top 45% of brain perfusion, red = 85% and white 93%. Healthy shows full even, symmetrical perfusion with most active area in cerebellum. Classic PTSD shows increased anterior cingulate, basal ganglia and thalamus perfusion. Classic TBI shows multiple areas of low perfusion seen on surface scans (top row). TBI and PTSD show both.

Fig 3 visually displays with 3-D rendered SPECT maps using Picker Odyssey Software (Eclipse Systems Inc., Branford CT) the different findings in TBI versus PTSD and in persons with both conditions. The first volume rendered row shows inferior underside surface rendered images. The second row shows intensity projection images in which white colors represent the top 8 percent of cerebral flow in that subject’s brain compared to their whole brain perfusion. A healthy control shows normal higher perfusion to the cerebellum. The PTSD subject shows increased perfusion in the brain—particularly in the frontal lobes. The TBI subject shows decreased perfusion throughout by comparison. The subject with both PTSD and TBI shows perfusion that is lower than the person with PTSD but higher than the subject with TBI.

## Discussion

The present study examines resting state and on-task rCBF differences which distinguish PTSD from TBI, either disorder from TBI+PTSD, and all three conditions from normal controls. When compared in a larger population with high psychiatric morbidity, TBI, PTSD and TBI+PTSD could be distinguished from non-TBI/non-PTSD with reasonable ROC characteristics which are similar across rest and task states, whether using quantitative or visual analysis.

Since visual analysis of resting state brain perfusion SPECT is a routinely performed and widely-available nuclear medicine procedure, the potential exists for the use of this test in clinical settings. Furthermore, the absence of any requirement for symptom-provocation, a commonly employed technique in functional imaging studies of PTSD, may make a resting state study more acceptable to individuals with active symptoms and to referring physicians. This investigation also uses the non-distressing Conners Continuous Performance Test for an activation task in all cases.

Compared to multiple severities of TBI—incurred primarily from blunt force—our findings showed increases in the limbic structures, cingulum, basal ganglia, insula, thalamus, prefrontal cortex and temporal lobes in subjects with PTSD. These results are consistent with the limited functional neuroimaging literature on PTSD. At baseline, both military and civilian PTSD subjects show increased perfusion in the caudate/putamen area, right temporal, orbitofrontal cortex, limbic regions, anterior cingulum, cerebellum, and medial prefrontal cortex [24–31, 94–96]. A recent meta-analysis showed that PTSD patients had significant activation in midline areas implicated in self-referential processing and autobiographical memory [75]. Peterson et al.’s recent survey takes a network-based approach to findings in 11 fMRI studies which met her quality threshold over the survey period, 2009 to mid-2013 [77]. They report a positive correlation between default mode network (DMN) connectivity in PTSD severity in five studies, negative in two. Similarly, the present data replicate SPECT findings in TBI. Specifically, hypoperfusion in the orbitofrontal cortex, temporal poles, and anterior cingulum are consistent with the most frequent findings in the TBI literature [8].

The symptoms of chronic TBI can often overlap with those of PTSD [2, 3, 32, 39, 48, 58]. About 15–19% of returning service members have probable mild TBI [34, 35], while an estimated 8–19% meet criteria for PTSD [2, 59, 97, 98]. The overlap of these two populations has been estimated at approximately 40% [34, 35, 39]. Neuropsychological testing has been unsuccessful in clearly differentiating these two disorders [99, 100]. These two overlapping populations have potentially different treatment requirements and different prognoses. For example, the treatments for PTSD may be harmful or, at best, not helpful in the case of TBI. The pharmacological treatments for PTSD, such as benzodiazepines and atypical antipsychotics [101, 102], can impede function or be dangerous in those who have TBI [3, 103, 104]. Similarly, antipsychotics are often prescribed for Veterans with PTSD [103, 105–107], but have been shown to impede recovery or be contraindicated in clinical studies and animal models of TBI [108, 109]. Other treatments for PTSD, such as transcranial magnetic stimulation [110], can be harmful in TBI due to induction of seizures [111]. Emerging treatments for TBI are more targeted and require an understanding of what portion of the individual’s brain is involved.

TBI is underappreciated as a contributing factor to the persistent symptoms experienced by service members, athletes [112, 113], and others who experience mild TBI [114, 115]. Differentiating TBI from PTSD is difficult based on symptoms alone or by neuropsychological testing. The treatment for TBI is considerably different from that for PTSD. Therefore, a specific and sensitive biomarker is needed that can readily distinguish TBI from PTSD [56, 59, 63].

The present study demonstrates a novel application of brain SPECT imaging to differentiate TBI from PTSD with sufficient sensitivity, specificity and accuracy to incrementally enhance clinical decision-making. The strengths of this work include the use of both resting state and concentration task scans from an objectively validated functional imaging modality, detailed quantitative analysis, and an extensively chacterized psychiatric population obtained across multiple sites. The large sample size, while a critical attribute, is further enhanced by the separate analysis of a carefully matched smaller cohort that still has a relatively large sample size.

This study also includes several potential limitations. First, this was a retrospective analysis, and we acknowledge that higher levels of evidence can be derived from either prospective studies or randomized clinical trials. Second, subjects in this study had varying degrees of injury severity. While this improves the overall generalizability of our results, future prospective studies investigating a particular class of injury severity (mild, moderate, severe) within a specific cohort (veteran, civilian) with a specific type of injury (blast, penetrating) will be prudent in validating these findings. Third, we did not account for the effect of socioeconomic status which is important as it is a risk factor for PTSD. And fourth, this dataset did not have accompanied structural imaging data, which would have provided useful information on hypoperfusion-associated atrophy, particularly in TBI.

## Conclusion

In summary, this is the first SPECT imaging study performed at rest and on-task demonstrating the ability to differentiate PTSD from TBI of varying degrees of severity in large patient cohorts with multiple comorbidities using both ROI and visual analysis. A clinically relevant level of sensitivity, specificity and accuracy was achieved. When compared to subjects with TBI, relative increases in perfusion were observed in PTSD in the limbic regions, cingulum, basal ganglia, insula, thalamus, prefrontal cortex and temporal lobes. These results suggest that TBI is associated with hypoperfusion while PTSD is associated with regional hyperperfusion, providing important insights regarding pathophysiological differences between the disorders. Replication of this work, even in smaller cohorts, would provide a solid basis for identification of biomarkers distinguishing TBI from PTSD, and has the potential to yield significant prognostic value in treating veteran, active military and civilian populations.
